# Histopathological and Ultrastructural Study of a Canine Langerhans Cell Tumour (Canine Cutaneous Histiocytoma)

**DOI:** 10.3390/cells13151263

**Published:** 2024-07-26

**Authors:** Isabel Pires, Paula Rodrigues, Anabela Alves, Filipe Silva, Carlos Lopes

**Affiliations:** 1 Animal and Veterinary Research Centre (CECAV), University of Trás-os-Montes and Alto Douro, 5000-801 Vila Real, Portugal; pavelar@utad.pt (P.R.); aalves@utad.pt (A.A.); fsilva@utad.pt (F.S.); 2 Department of Veterinary Sciences, University of Trás-os-Montes e Alto Douro (UTAD), 5000-801 Vila Real, Portugal; 3 School of Medicine and Biomedical Sciences, University of Porto, 4099-002 Porto, Portugal

**Keywords:** canine cutaneous histiocytoma, skin tumours, dog, histopathology, ultrastructural, TEM, Langerhans cells, canine, dendritic cells

## Abstract

Canine cutaneous histiocytoma (CCH) represents a significant proportion of dog skin tumours, often manifesting as the most common neoplastic skin condition in young animals. Predominantly affecting dogs under four, these tumours appear primarily as solitary lesions that may regress spontaneously. This study, conducted over five years at the University of Trás-os-Montes e Alto Douro, involved a detailed histopathological and ultrastructural examination of 93 CCH cases. Histologically, these tumours showed distinct patterns of lymphoid infiltration, which contributed to their classification into four groups based on the inflammatory response and histological architecture. Most tumours displayed signs of epidermal invasion and frequent mitotic figures, with necrosis present in over half of the cases. Ultrastructurally, the neoplastic cells were characterised by pleomorphism, abundant organelles, and adherens-type junctions. This study offers significant insights into the pathophysiology and morphological characteristics of CCH, underscoring the importance of detailed histological and ultrastructural analysis in accurately diagnosing and understanding this common canine tumour.

## 1. Introduction

Canine cutaneous histiocytoma (CCH) is a common benign tumour that arises from Langerhans cells in the epidermis of dogs. Despite its rapid growth and high mitotic index of CCH, which may suggest malignancy, CCH rarely metastasises and is known for its propensity to undergo spontaneous regression [1,2,3].

CCH represents approximately 19% of skin and subcutaneous tissue tumours in dogs, with a higher prevalence in young animals. Although it can occur in dogs of any age, 60% to 70% of cases involve animals under four years old. They are more frequent in the external nasal region, ears, limbs, and scrotum [4,5,6,7,8].

The typical clinical manifestation of CCH includes solitary, rapidly growing, erythematous, elevated, button-like alopecic nodules that can range from 0.5 to 1.5 cm in diameter but may grow up to 4 cm. Multiple histiocytomas can occur synchronously or sequentially, especially in the Shar-Pei breed [1,7,9,10,11].

CCH cells express CD1 in cases where normal Langerhans cells also express it, and diffusely in all cases, CD1b, CD1c, CD11a, CD11c, MHC class II, CD44 and E-cadherin. In most cases, there is immunoreactivity for CD11b, CD49d-like (VLA-4), and ICAM-1. Tumour cells do not express CD11d, Thy-1, or CD4 [2,12,13]. Expression of Multiple Myeloma Oncogene 1/Interferon Regulatory Factor 4 (MUM1/IRF-4) is also described [14].

Spontaneous regression, occurring a few weeks or months after their appearance, is the natural progression of this neoplasia [15]. Although this phenomenon is observed in various human and animal tumours, the mechanisms behind CCH’s regression are not fully understood. This regression is often linked to factors such as reduced tumour cell proliferation, enhanced apoptotic activity [16], lack of VEGF-A [17], changes in the expression of extracellular matrix metalloproteinases, such as MMP-9 [18], and a complex interplay of immune responses. This includes the peripheral migration of MHC class II molecules [15,19], a CD206 phenotype change, an increase in CD4 T lymphocytes and CD8 T lymphocytes and a rise in Th1 pro-inflammatory cytokines such as IL-2, tumour necrosis factor-α (TNF-α), interferon-γ (IFN-γ), up-regulation of the nitric oxide synthase (iNOS) expression and M1 macrophages [20,21].

CCH resembles human Langerhans cell histiocytosis (LCH), though LCH exhibits different clinical behaviours in humans compared to dogs. CCH’s ability to regress spontaneously has positioned it as a valuable model for studying the elusive pathology of LCH in humans, providing insights into potential triggers and pathways of tumour regression across species [19].

This study aims to contribute to canine cutaneous histiocytomas’ histopathological and ultrastructural characterisation.

## 2. Materials and Methods

### 2.1. Material Collection and Light Microscopy Study

In this study, 93 tumours were analysed at the Histology and Pathological Anatomy Laboratory of the University of Trás-os-Montes e Alto Douro. These samples were received from various veterinary clinics across different districts of Portugal. Data regarding the animal identification (breed, gender, age), anatomical localisation, and lesion size were recorded whenever possible.

The collected material was fixed in 10% commercial formalin, processed, and embedded in Histoplast^®^-Shandon^®^ (Thermo Fisher Scientific, Kalamazoo, MI, USA) paraffin for histopathological evaluation. Sections of 3 µm thickness were prepared and stained with hematoxylin–eosin. Systematic observation and histological classification of the tumours were performed according to the WHO criteria [22].

Two independent observers (I.P. and A.A.) diagnosed and classified histiocytomas using the same criteria. The slides were evaluated and described under a Nikon FXA^®^ microscope. The following parameters were also assessed and categorised: epidermis covering the tumour, ulceration, nuclear pleomorphism, mitotic count, stroma, necrosis extension and localisation, and the presence of multinucleated cells. The condition of the epidermis covering the tumour was noted as normal, with regular hyperplasia, irregular hyperplasia, pseudocarcinomatous hyperplasia, or ulcerated (absent over the entire tumour area). Ulceration was categorised as absent (0), small with microscopic dimensions (1), moderate but not affecting the whole surface of the tumour (2), and extensive, involving the epidermis covering the entire tumour area (3). The mitotic count, or the number of mitoses per high-power field (40×, counting an average of 10 fields in different tumour regions), was recorded as less than 2 mitoses, 2 to 5 mitoses, and more than 5 mitoses. The stroma was classified as scarce (1), moderate (2), or abundant (3).

Necrosis was evaluated for its extent as absent (0), scarce (1), moderate (2), or extensive (3), and its localisation was noted as peripheral or diffuse (observed at the periphery and centre of the tumour).

The presence of multinucleated cells was recorded as absent (0) or present (1).

According to Cockerell and Slauson, the tumours were categorised into four groups based on the relative amount and distribution pattern of lymphoid inflammatory infiltrate. Group I included lesions with minimal inflammatory infiltrate at the base. Group II included lesions with moderate nodular lymphoid infiltrate at the periphery. Group III tumours exhibited abundant infiltration up to the centre of the lesion. In contrast, Group IV lesions showed infiltrate that exceeded the histiocytic cells and extended to the epidermal surface [23].

Images were captured using a DXM1200 digital camera (Nikon Instruments Inc., Melville, NY, USA) attached to an Eclipse E600 microscope from the same manufacturer.

For statistical analysis, chi-square tests (χ^2^) were conducted using IBM SPSS Statistics (IBM Corporation, Armonk, NY, USA) version 21, to explore potential associations between variables. The results are presented as absolute and relative frequencies. Significance levels were set at *p* < 0.05.

### 2.2. Electron Microscopy Study

Ultrastructural characterisation was carried out using transmission electron microscopy on material fixed in buffered formalin, following the methods established at the Institute of Histology and Embryology of the Faculty of Medicine of Coimbra.

Tumours for the ultrastructural study were selected based on formalin fixation time and available material. Twenty cases from Groups I and II were used.

The tumour tissue samples and the transition zone between the epidermis and dermis were meticulously processed to ensure accurate histological analysis. Initially, the material was cut into small fragments approximately 1 mm^3^, strategically representing different areas of the tumour and the adjacent epidermal–dermal junction. These samples were then submerged in a dual-fixative solution of 4% formaldehyde and 0.5% glutaraldehyde in a 0.1 M phosphate buffer at a neutral pH of 7.4. This step was carried out for 2 h at a controlled temperature of 4 °C, ensuring optimal tissue architecture and molecular integrity preservation.

Following the initial fixation, the samples underwent post-fixation in an aqueous solution containing 1% osmium tetroxide (O_S_O_4_) for 1.5 h at 4 °C, enhancing the sample’s contrast and detail for electron microscopy.

The samples were dehydrated for 10 min in a graded series of alcohol concentrations: 50%, 70%, and 95% alcohol solutions. To completely remove any traces of water, the tissue was passed twice through absolute alcohol for 10 min each.

After dehydration, the samples were prepared for embedding and initially impregnated twice with propylene oxide for 15 min each to facilitate infiltration. The tissue was then placed in a mixture of propylene oxide and epoxy resin at a ratio of 3:1 for 2 h with agitation, followed by a 1:1 mixture overnight to ensure thorough penetration of the embedding medium.

Finally, the samples were embedded in pure epoxy resin for 4 h and positioned using the flat embedding method, which is critical for orienting the fragments according to the representation of the dermo-epidermal junction.

The resin was polymerised in an oven at 58 °C for 32 to 48 h, solidifying the resin around the tissue fragments. The blocks were then trimmed using a diamond drill in the Reichert^®^ TM 60 unit (AMETEK, Inc., Depew, NY, USA) to obtain a precise cutting surface of approximately 1 mm^2^.

For microscopy, semi-thin sections ranging from 1 to 1.5 mm in thickness were collected on slides, dried, adhered on a heated plate, and stained with 0.025% toluidine blue in a sodium borate solution at 65 °C for 30–60 s, followed by differentiation in distilled water.

The previously trimmed blocks were further sectioned into ultra-thin slices between 450 and 700 nm. These sections were collected on copper/rhodium grids with a 300 mesh Taab^®^ HR25 (Taab Laboratories Equipment Ltd., Aldermaston, Berks, UK) and air-dried at room temperature. Contrast staining was applied using 2% uranyl acetate solution and 0.4% lead citrate, enhancing the visibility of cellular structures under the microscope.

All solutions and rinses used ultra-pure water. Sample observation was carried out using a Jeol^®^ 100S transmission electron microscope (JEOL Ltd., Tokyo, Japan), and photographic documentation was performed on Kodak Electron Microscope Film 4489^®^ (Eastman Kodak Company, Rochester, NY, USA).

## 3. Results

### 3.1. Clinical Data

The tumours formed nodules and exophytic formations, often presenting as single, button-like, alopecic, and frequently ulcerated lesions. According to the information collected during the anamnesis, the progression of the lesion was rapid, with excision occurring 1 week to 2 months after its appearance. In one case, the progression lasted 6 months.

Of the tumours studied, 39 (42%) occurred in females and 52 (58%) in males. In two cases, the gender of the animal could not be determined. At the time of surgery, the mean age was 2 years, ranging from 2 months to 12 years. Most tumours occurred in animals aged 2 years or younger (n = 62; 81.58%). Only 10 animals (13.16%) were aged 4 years or older. In 17 cases, the age of the animal could not be determined.

Histiocytomas were more frequently found in purebred animals (n = 83; 89.25%). In our study of canine cutaneous histiocytoma, we observed that Boxers were the most frequently affected breed with 26 cases (28.0%), followed by Cocker Spaniels with 14 cases (15.1%), and dogs of indeterminate breed with 10 cases (11.2%). Rottweilers accounted for nine cases (9.7%), and Siberian Huskies had five cases (5.4%). Pit Bulls and Labrador Retrievers each had four cases (4.3%). German Shepherds, Poodles, Dobermanns, Portuguese Pointers, and Estrela Mountain Dogs had two cases (2.2%). Several breeds had one case (1.1%) each, including Bull Terriers, Chow-Chows, Dalmatians, Great Danes, Golden Retrievers, Rhodesian Ridgebacks, Miniature Pinschers, Irish Setters, Shar Peis, Staffordshire Terriers, and Dachshunds (Teckels).

The average size of the tumour lesions was 1.4 cm, ranging from 0.4 to 3 cm in diameter. In 38 cases (40%), tumours had a diameter greater than 1.4 cm. In 90% of the cases, the lesions were 2 cm in diameter or smaller. Regarding anatomical localisation, 35 HCC (39.77%) were located on the head, particularly on the auricular pavilion (n = 16). A total of 33 cases (37.5%) affected the limbs, mainly on the distal extremity, including the toes. The remaining tumours were located in the cervical (9.1%), thoracic (n = 7; 8%), and abdominal (n = 5; 5.7%) regions. In five cases, we could not obtain information about the location of the lesion.

The animals were followed clinically for 2 years, and according to the information obtained, 2 years after excision, no cases recurred locally or metastasised at a distance.

### 3.2. Light Microscopy Study

**Figure 1:**
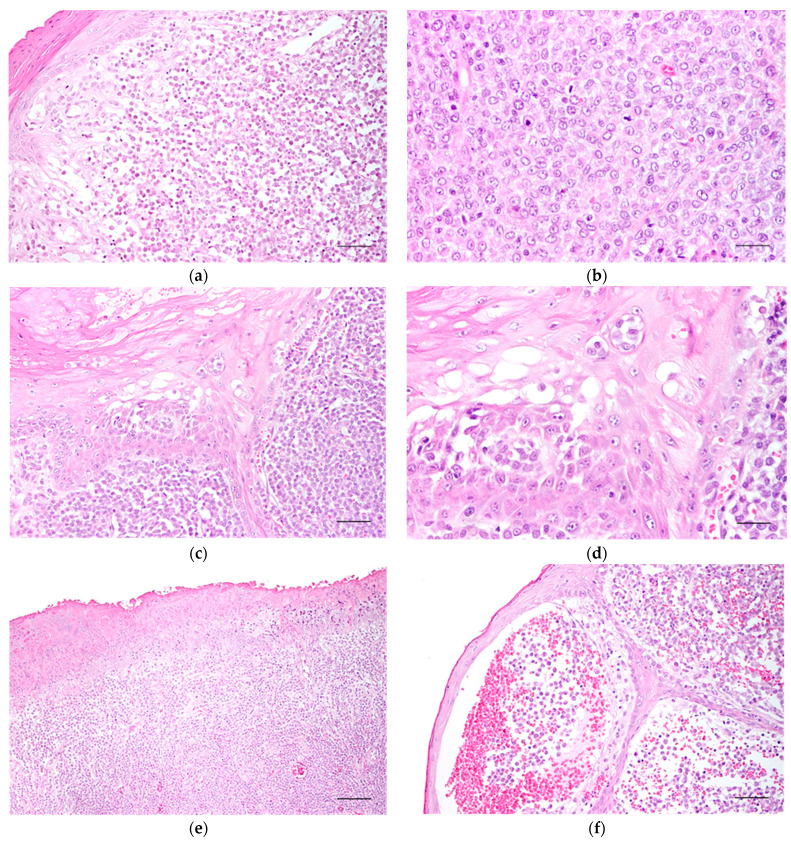

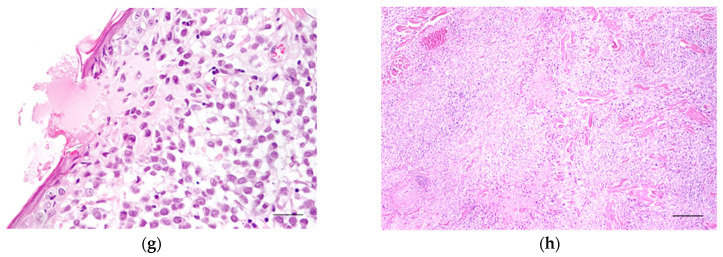
Light microscopy images of canine cutaneous histiocytoma. (**a**) Tumour cells occupy the superficial dermis. Scale bar = 60 μm; (**b**) cells with indistinct contours and vesiculated nuclei with occasional nucleolus. Scale bar = 30 μm; (**c**) epidermal “invasion” by the neoplastic cells of the canine cutaneous histiocytoma. Scale bar = 60 μm; (**d**) epidermal “invasion” by neoplastic cells (higher magnification). Scale bar = 30 μm; (**e**) extensive ulceration of the epidermis. Scale bar = 120 μm; (**f**) pseudocarcinomatous hyperplasia of the epidermis. Scale bar = 60 μm; (**g**) small epidermal ulcer in the epidermis covering the CCH. Scale bar = 30 μm; (**h**) moderate areas of necrosis at the periphery and centre of the tumour. Scale bar = 120 μm.

Canine cutaneous histiocytoma presented as an expansively growing neoplasm composed of mononuclear cells that exhibit moderate pleomorphism and are distributed up to the dermo-epidermal junction (Figure 1a). The cytoplasm of the neoplastic cells varied from scant to abundant and was either acidophilic or occasionally vacuolated. The nuclei were voluminous, generally eccentric, and exhibited hyperchromasia or a vesicular appearance with peripheral hyperchromasia. They were oval, round, or kidney-shaped, with prominently visible nucleoli (Figure 1b).

Mitotic figures were frequently observed, ranging from 0 to 12 per high-power field. Signs of individual cell death were commonly noted throughout the samples. The tumour stroma was typically sparse (77.4% of cases) and exhibited low vascularity, with no apparent increase in blood vessels compared to the normal dermis. Collagenisation was occasionally observed in some areas.

Epidermal “invasion” by tumour cells was frequently observed (Figure 1c,d). The overlying epidermis exhibited varying characteristics: in seven cases (7.5%), it appeared normal; in 32 cases (58%), it was ulcerated across its entire extent (Figure 1e); and in 35 cases (37.6%), it exhibited pseudocarcinomatous hyperplasia with features such as acanthosis, hypergranulosis, and hyperkeratosis (Figure 1f). At the locations of the epidermal papillae, the tumour surfaced through the epidermis with a blurred demarcation between the two zones. In eight lesions (8.6%), superficial fibrosis areas were observed, separating the epidermis from the tumour.

Ulceration was present in 83 (89%) of the preparations; however, in 20 cases (21.2%), the ulcers were small and only visible under a microscope (Figure 1g). Typically, a superficial neutrophil infiltrate was associated with the ulceration. Around the ulcerated regions, a reduction in the number of epidermal layers was observed, along with a thinning of the germinative and spinous strata. The basal membrane was absent in these areas, indicating epidermal invasion by tumour cells.

In over half of the cases (63.3%), necrosis was moderate and located at the basolateral periphery and the centre of the tumour. These areas were characterised by a hypocellular central region with acidophilic amorphous debris and occasionally pyknotic nuclear remnants (Figure 1h). Necrosis was absent in 33 cases (35.5%).

**Figure 2:**
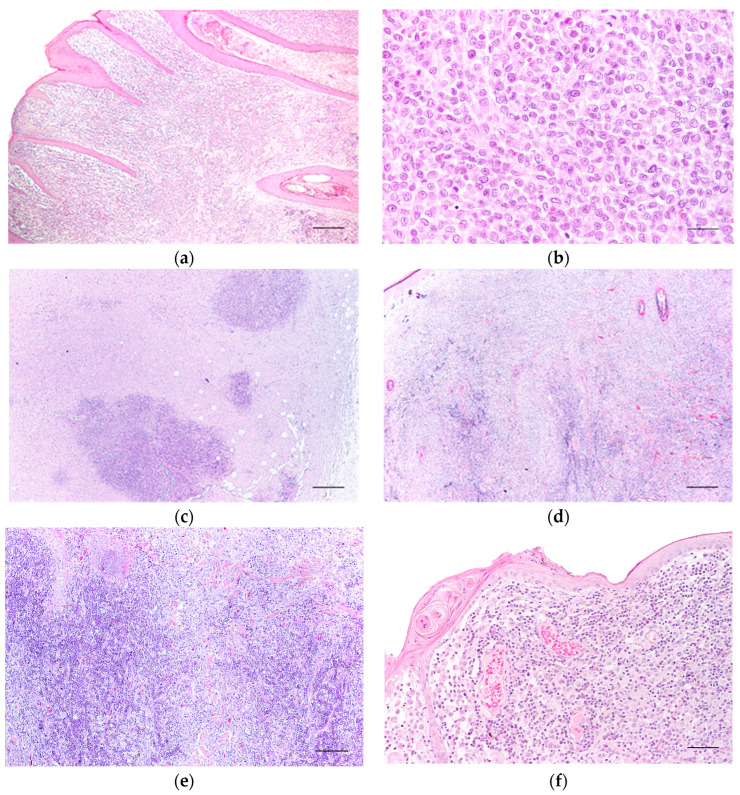

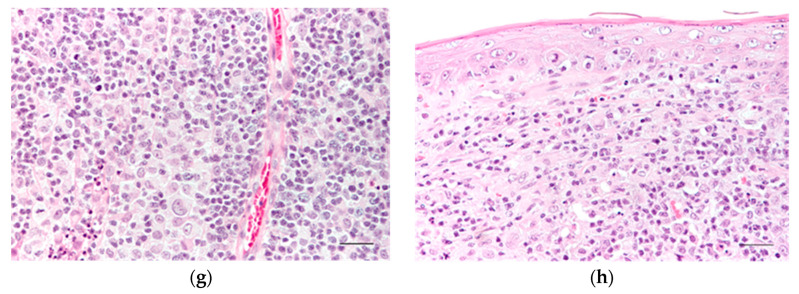
Light microscopy images of canine cutaneous histiocytoma. (**a**) Canine cutaneous histiocytoma from histological Group I; the tumour cells occupy the superficial dermis. Lymphoid infiltrate at the periphery of the lesion. Scale bar = 300 μm; (**b**) detail of the previous image. Scale bar = 30 μm. (**c**) Canine cutaneous histiocytoma from histological Group II; the lymphoid infiltrate forms follicles at the periphery of the tumour. Scale bar = 300 μm. (**d**) Canine cutaneous histiocytoma from histological Group III; the lymphoid infiltrate is abundant in the centre and periphery of the tumour. Scale bar = 300 μm; (**e**) detail of the previous image. Scale bar = 60 μm. (**f**) Canine cutaneous histiocytoma from histological Group IV; the lymphoid infiltrate is abundant and reaches the surface of the tumour. Tumour cells are scarce. Scale bar = 60 μm; (**g**) CCH from Group IV; the lymphoid infiltrate is more abundant than the tumour cells. Note the polygonal shape of the cells. Scale bar = 30 μm; (**h**) CCH from Group IV, showing scarce tumour cells, abundant lymphocytes, and fibroblast proliferation at the dermo-epidermal junction. Scale bar = 60 μm.

Group I (Figure 2a) tumours exhibited minimal lymphoid inflammatory infiltrate, which, when present, was confined to the periphery of the lesion. The neoplastic cells were uniform, with finely granular and acidophilic cytoplasm. Nuclei were round or oval, occasionally with nucleoli, and frequent mitotic figures ranged from 2 to 12 per high-power field (Figure 2b). Multinucleated cells were observed in one case. At the tumour periphery, the cells formed nests or cords with well-defined contours, sometimes separated by superficial oedema. Deeper in the tumour, the proliferation was solid, and the cells exhibited sparser cytoplasm with indistinct cellular contours. The tumour stroma was sparse. The epidermis was often hyperplastic, with pseudocarcinomatous hyperplasia in more than half of the cases. Ulceration was present in most cases (n = 13; 86.7%), typically small in size. Necrosis, when present (n = 6; 40%), was generally sparse and located at the periphery of the tumour.

Neoplasms in Group II (Figure 2c) displayed moderate lymphoid infiltration at the tumour’s periphery, organised into nodular formations, sometimes with an apparent organoid structure centred by a blood vessel. The morphology of the neoplastic cells was identical to that described in the previous group, with multinucleated cells observed in three cases. The epidermis was hyperplastic in 10 cases (66.6%), showing irregular or pseudocarcinomatous hyperplasia or extensively ulcerated (n = 5; 33.3%). Ulceration was present in almost all cases (n = 14; 93.3%), with a predominance of extensive ulcers (n = 8; 53.3%). Necrosis was observed in 66.7% (n = 10) of the lesions, mainly sparse to moderate at the basolateral periphery.

Tumours in Group III (Figure 2d,e) exhibited abundant lymphoid infiltration, capable of forming nodular structures both at the periphery and the centre of the tumour. Neoplastic cells constituted a smaller proportion of the total cell population. The nuclei were less dense, and the nucleolus was more frequently visible. The cytoplasm was occasionally vacuolated, and cells sometimes appeared polygonal. Solid areas seen in Groups I and II tumours were observed at the centre and surface of the tumour but were smaller, located amid intense inflammatory infiltration. The stroma was scarce, except in seven cases (16.7%). Sometimes, fibroblast proliferation was observed at the apical surface of the lesion, separating it from the epidermis, which was normal in these areas. The epidermis was generally hyperplastic (n = 24; 57.2%) and frequently ulcerated (n = 37; 88.1%), with intense infiltration by neutrophils, reaching deep areas of the tumour in two cases. In 14 cases (33.3%), the ulcer covered the entire surface of the neoplasm. Necrosis was evident, moderate to extensive (n = 25; 59.6%), both in the centre and at the base of the tumour. Multinucleated cells were observed in six cases (14.3%).

Group IV included tumours with very intense inflammatory infiltration (Figure 2f). This infiltration was observed from the tumour margins to the dermo-epidermal junction, occupying an area larger than the neoplastic cells (Figure 2g). The neoplastic cells, with well-defined cellular contours, appeared individualised and not in solid areas located from the surface into the lesion. Some cells, polygonal in shape, had pale cytoplasm and a pleomorphic, round to oval, and indented nucleus. In contrast, others appeared degenerated with eosinophilic cytoplasm and a small, eccentric “C-shaped” nucleus. Multinucleated cells, primarily binucleated, were frequent. The tumour stroma was moderate. Ulceration was present in almost all cases (n = 21; 90.5%), and the epidermis, when present, was generally hyperplastic. In two cases (9.5%), the epidermis appeared normal. In certain areas, the epidermal ridges were non-existent, and a single layer of cells represented the different epidermis layers. Fibrous tissue separated the epidermis from the remaining tumour (Figure 2h). Areas of necrosis observed in 50.0% (n = 10) of the tumours were moderate or extensive. In cases where necrosis was not evident, areas of fibrosis were observed among the inflammatory infiltrate.

Table 1 resumes the histopathological parameters evaluated.

### 3.3. Associations between Clinical and Histopathological Parameters

There were no significant associations among the clinical variables studied, except for size and location (*p* = 0.016) of the tumour. Tumours larger than 2 cm were more frequent in the neck and limbs.

We found statistically significant associations between clinical and histological variables. Age and ulceration were significantly associated (*p* = 0.033); younger animals had more extensive ulcers, although there were also non-ulcerated neoplasms, while in older animals, the neoplasms were always ulcerated. The presence of multinucleated cells was significantly related to tumour location (*p* = 0.036); abdominal tumours mostly (80%) contained multinucleated cells, whereas tumours in other locations predominantly lacked these cells. Additionally, fibrosis was significantly associated with tumour location (*p* = 0.019); tumours with superficial fibrosis were generally located in the head. Sex and mitotic index also showed a significant association (*p* = 0.018), with male animals having tumours with a higher mitotic index than females. Lastly, tumour size and tumour stroma were significantly related (*p* = 0.019); tumours with scarce stroma were always smaller than 2 cm.

Regarding histological groups, tumour size was the only clinical variable that showed significant differences among the histological groups (*p* = 0.042). In Group I, 26.7% of the cases had tumours larger than 2 cm. In Group II, this proportion was also 26.7%. In Group III, 19.1% of the samples had tumours larger than 2 cm. In Group IV, all tumours were smaller than 2 cm.

Among the following histological variables, there were statistically significant differences between the distribution and extent of necrotic areas (*p* < 0.0001), with extensive necrosis generally being diffuse; ulceration and the presence of multinucleated cells (*p* = 0.045), where tumours with multinucleated cells mainly had larger ulcers; and the mitotic index with other histological variables such as stroma (*p* < 0.0001), superficial fibrosis (*p* = 0.043), and multinucleated cells (*p* = 0.026). Tumours with a mitotic index lower than 2 predominantly had moderate stroma. None of the tumours with more than five mitoses per high-power field exhibited superficial fibrosis. Multinucleated cells were more frequent in tumours with fewer than five mitoses.

There were significant differences across the histological group of ulceration (*p* = 0.026), the distribution of necrosis in the tumour (*p* = 0.0002), the presence of multinucleated cells (*p* = 0.003), the mitotic count (*p* = 0.0001) and the stroma (*p* = 0.0001).

Extensive ulceration was more frequent in Groups III and IV. Necrosis was generally absent or peripheral in the tumours of Groups I and II and diffuse in those of Groups III and IV. Multinucleated cells were more common in Group IV tumours. The mitotic index was higher in Group I and II, decreased in Group III, and was generally less than 2 in Group IV. The stroma was scarce in histiocytomas classified in Groups I and II and abundant in some tumours in Group III and more than half of Group IV.

### 3.4. Ultrastructural Study

**Figure 3:**
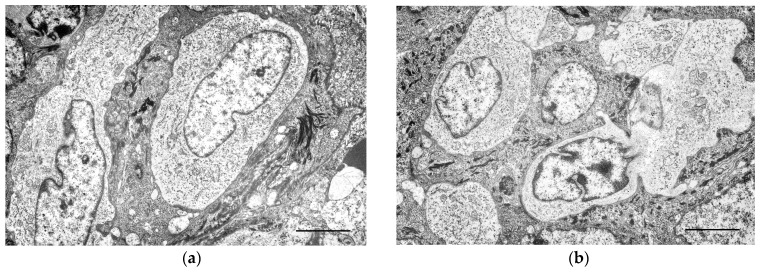

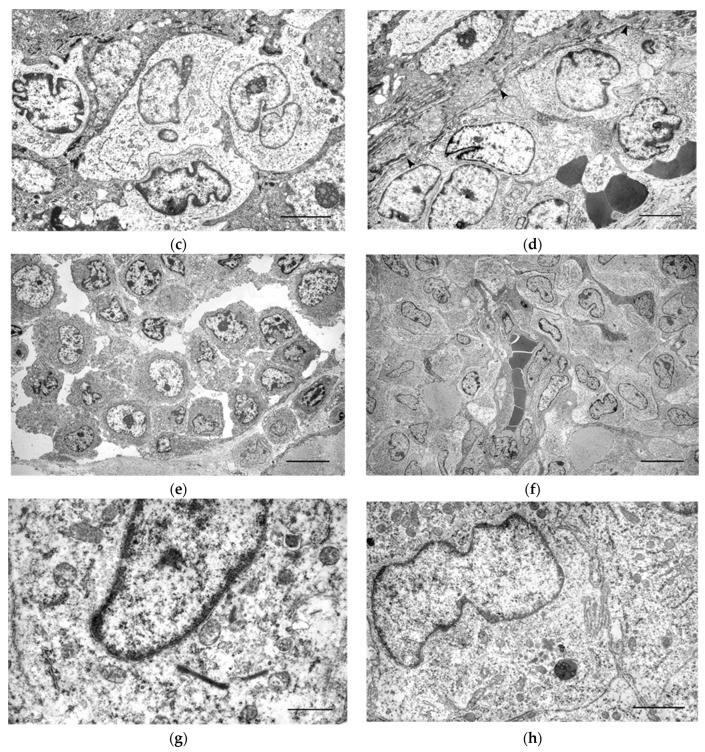
Transmission electron microscopy images. (**a**) Epidermis, showing Langerhans cells (LC). Scale bar = 4 μm; (**b**) LC in the epidermis adjacent to the canine cutaneous histiocytoma, displaying extensions between keratinocytes. Scale bar = 4 μm; (**c**) image of epidermal ridges, where groups of LC can be observed. Scale bar = 4 μm; (**d**) intimate relationship of tumour cells with the dermo-epidermal junction. Scale bar = 5 μm.; (**e**) in the superficial region of the CCH, the tumour cells are loosely organised. Scale bar = 10 μm; (**f**) in deeper regions, the tumour cells form solid areas with scant stroma. Note the irregular nuclei, sometimes with a prominent nucleolus. Scale bar = 10 μm; (**g**) image of the tumour cell cytoplasm, where cytoplasmic organelles can be observed. Note rod-shaped structures. Scale bar = 1 μm; (**h**) tumour cell cytoplasm, showing pleomorphic vesicles, some with a multivesicular appearance. Scale bar = 2 μm.

In the ultrastructural study for Group I and II CCH, the epidermis was hyperplastic, with increased layers in the spinous, granular, and corneous layer, forming irregular crests. Cells identical to the tumour cells were observed in the epidermis with a suprabasal location, interpreted as Langerhans cells (Figure 3a,b). These were clear cells, characterised by abundant rough endoplasmic reticulum and a moderate number of mitochondria, and distinguished from keratinocytes by the absence of tonofilaments and desmosomes. In the hyperplastic epidermal crests, the number of Langerhans cells was increased (Figure 3c).

Cell proliferation was observed extending from the superficial dermis to the dermo-epidermal junction (Figure 3d). Near the dermo-epidermal junction, the tumour cells were organised into loosely compacted nests or cords (Figure 3e). The cells adopted a more solid pattern in deeper regions, with the extracellular matrix being nearly imperceptible (Figure 3f). The cells were monomorphic or displayed moderate pleomorphism, especially in the superficial areas, averaging 10.5 µm to 14.6 µm in size.

The nuclei were pale, generally central, round or reniform, sometimes lobulated, and measured 6.5 µm to 10 µm on its central axis. The nucleolus was voluminous, dense, and either central or eccentric.

The cytoplasm was clear and contained various organelles (Figure 3g). Ribosomes were numerous, both free and associated with the endoplasmic reticulum. The rough endoplasmic reticulum was represented by multiple tubules and cisternae, sometimes dilated. The mitochondria, round to oval, were frequent. The Golgi apparatus was less evident.

Pleomorphic vesicles of variable size, generally round to oval and rarely rod-shaped, were observed (Figure 3h). Their content was electron-dense, granular, and sometimes multivesicular, resembling secondary lysosomes. Some vesicles had irregular contours with electron-dense areas.

**Figure 4:**
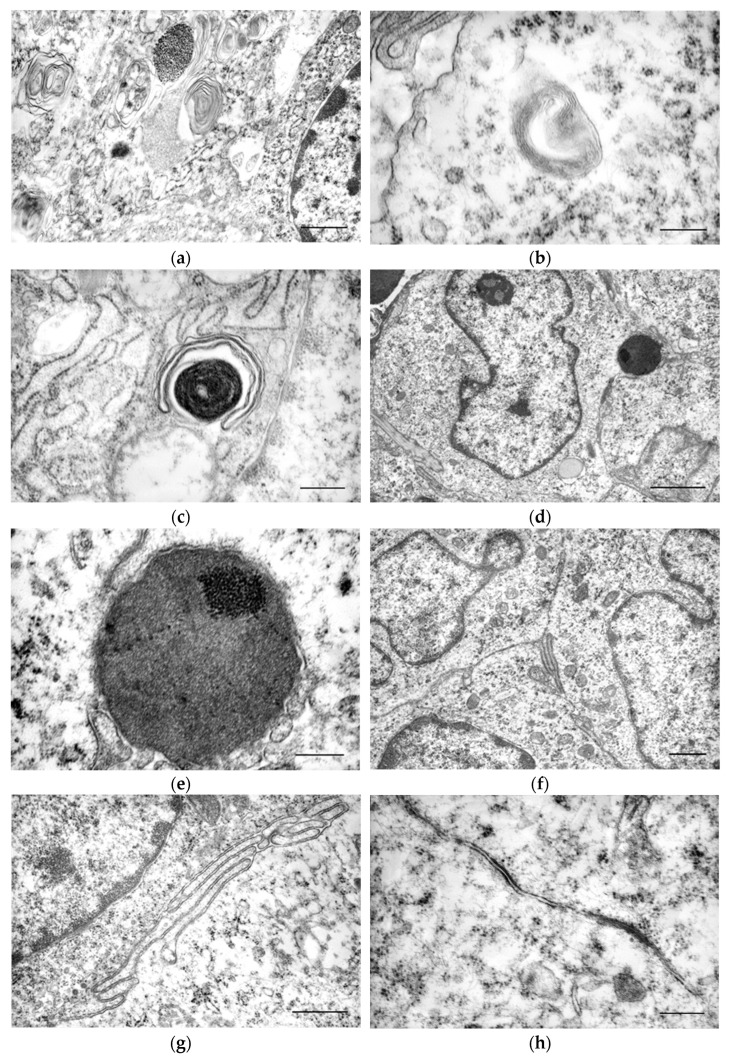
TEM images. (**a**) Multivesicular and multilamellar structures in the cytoplasm of tumour cells. Scale bar = 1 μm; (**b**) detail of the multilamellar structure with concentric arrangement. Scale bar = 400 nm; (**c**) cytoplasm of a tumour cell of the CCH, showing multilamellar structure with a dense central arrangement. Scale bar = 500 nm; (**d**) image of the CCH, noting a cell with condensation of a paracrystalline aspect. Scale bar = 2 μm.; (**e**) detail of the previous image. Scale bar = 400 nm; (**f**) image obtained from a deep region of the CCH, where interdigitations of the cytoplasmic membrane between neighbouring cells are noticeable. Scale bar = 1 μm; (**g**) interdigitations of the cytoplasmic membrane between neighbouring cells are noticeable. Scale bar = 800 nm; (**h**) adherens-type cell junctions between neoplastic cells. Scale bar = 200 nm.

In some cells, concentric multilamellar electron-dense structures with a filamentous appearance were present—myelin-like inclusions with various configurations of the lamellar arrangement (Figure 4a,b), and some paracrystalline condensations were also observed (Figure 4c,d,e).

In the deeper regions of the neoplasm, where the cellular density was higher, interdigitations of the cytoplasmic membrane were frequently observed between neighbouring cells (Figure 4f,g), sometimes accompanied by adherens-type intercellular junctions (Figure 4h).

Within the neoplastic population, mitotic figures were frequent. A large number of cells with various morphological alterations characteristic of the apoptosis process were observed, such as peripheral condensation of nuclear chromatin, irregular nuclear envelope, dilatation of the endoplasmic reticulum, reduction in cell volume, modifications in the cytoplasmic membrane with bubble-like projections, cellular fragmentation into numerous apoptotic bodies, and phagocytosis of apoptotic bodies.

In addition to tumour cells, dendritic cells of the dermis, neutrophils, macrophages, lymphocytes, mast cells, and fibroblasts were also observed. The stroma was generally sparse, with a notable absence of normal collagenous dermis, and in some cases, images suggestive of collagenolysis were observed. Blood vessels were rare.

## 4. Discussion

Canine cutaneous histiocytoma (CCH) is a common tumour in young dogs [24,25,26] and is often associated with spontaneous regression. Characterised by the proliferation of histiocyte-like cells, these cells occupy the dermis and sometimes invade the dermo-epidermal junction. Immunophenotyping studies and transmission electron microscopy have shown that CCH originates from epidermal dendritic cells—Langerhans cells [2,4,27].

To characterise canine cutaneous histiocytoma (CCH), we analysed 93 cases from various veterinary clinics. While the tumours were primarily unique, multiple histiocytomas have been reported in some cases [2,9,13,28]. Recently, it has been proposed that these lesions could be included in the broader category of Cutaneous Langerhans Cell Histiocytosis (CLCH), which encompasses a range of diseases where multiple skin lesions are consistently present. Occasionally, metastasis to lymph nodes and other systems can occur [29].

In our study, tumours were more frequently observed in males and young animals and generally smaller than 2 cm, aligning with other researchers’ findings [2,15,23,30].

Cutaneous canine histiocytoma’s (CCH) distribution across breeds was quite broad. A study by Mulle suggests a predisposition in various pure breeds, particularly in Boxer and Cocker Spaniel breeds [30], observations that we also noted.

The most frequent locations for CCH were the head (mainly the ears, followed by the lips and the external nasal region) and the limbs, particularly the distal regions, including the digits, consistent with the work of other authors [30]. The higher incidence of lesions in these areas may be due to a combination of factors. Firstly, these areas are more exposed to sunlight. Following irradiation, there is an initial decrease in the number of Langerhans cells and co-stimulation molecules (B7), but their numbers eventually increase and then decrease again [31]. This fluctuation in Langerhans cell numbers might influence the development of CCH.

Additionally, the extremities, including the digits, are easily accessible to ectoparasites, and the head is vulnerable to insect bites. These processes trigger an immune response that could contribute to the genesis of CCH. Moreover, owners notice lesions in the more affected areas more easily than those in ventral locations. The thickness of the skin may also play a role in the localisation of lesions, as it varies with breed and anatomical location. The skin is thicker on the dorsal side of the head and the cervical region, thinner on the abdomen, and even thinner in the inguinal and axillary areas. On the limbs, skin thickness decreases from the proximal to the distal extremities [32]. This could explain the occurrence of lesions on the head, particularly on the ears, lips, and external nasal region, as well as on the digits of the limbs.

Concerning clinical parameters, there were statistically significant differences in tumour size (*p* = 0.042) among the different histological groups. Tumours in Groups III and IV presented smaller dimensions, likely due to regression, resulting in reduced neoplastic cell mass.

Microscopically, canine cutaneous histiocytoma is an expansively growing neoplasm of moderately pleomorphic mononuclear cells distributed up to the dermo-epidermal junction, with frequent mitotic figures, variable necrosis, sparse stroma, occasional epidermal invasion, and a significant presence of ulceration and lymphoid infiltrate.

Analysing the histopathological features across histological groups based on lymphoid inflammatory infiltrate, the results suggest a multifaceted nature of histiocytoma regression involving ulceration, necrosis, mitotic activity, stromal development, and multinucleated cell presence. Interestingly, ulceration increased with the histological group stage. Most tumours were ulcerated, and the severity of ulceration increased with the tumour stage, suggesting a relationship between ulcer size and lymphoid infiltrate. This finding supports the idea that ulceration may be an early event in the regression dynamics of histiocytomas.

Intratumoural necrosis varied significantly across the groups. Groups II and III exhibited more extensive necrosis than Groups I and IV, where necrosis was less frequent or absent. The distribution pattern of necrotic areas also differed, beginning at the periphery in the early stages and extending to the centre in later stages. This pattern may reflect the progression of cellular death and the immune response within the tumour microenvironment. The presence of necrosis in advanced groups, followed by its reduction in Group IV, suggests that necrosis is associated with tumour regression.

The mitotic count also showed significant differences between the histological groups. It was highest in Groups I and II, indicative of active cell division, and decreased in Groups III and IV, where it was generally lower than two. This reduction in mitotic index aligns with the regression phase of histiocytomas, supporting the hypothesis that regression is associated with decreased cellular proliferation. However, studies utilising Ki-67 and TUNEL indicate that an imbalance between cell proliferation and apoptotic cell death, rather than solely a decrease in cell proliferation or an increase in apoptosis, is a key factor driving CHH regression [16]. Given that none of the tumours recurred, it is not possible to associate them with increased aggressiveness.

This study found a significant correlation between the histological group and the amount of stromal tissue. Tumours in Groups III and IV had a moderate-to-intense lymphoid infiltrate with substantial stromal tissue, while Groups I and II had scant stroma. This association indicates that the development of stromal tissue may be related to the reduction in tumour mass and the histological group, implying a role in the resolution process.

Multinucleated cells were more frequently observed in tumours from Group IV. Multinucleated cells in higher stages may indicate advanced cellular changes and immune interactions within the tumour microenvironment.

In the ultrastructural study, the cells exhibited abundant mitochondria, ribosomes, rough endoplasmic reticulum, a scarcely evident Golgi apparatus, pleomorphic vesicles, multilamellar bodies, and paracrystalline structures. These findings are consistent with previous studies [3,4,33], although there was variation in the relative amount of cytoplasmic organelles. Birbeck granules were not observed, as expected, since normal epidermal Langerhans cells in dogs do not present these structures [32]. Apoptotic images were frequent, though not mentioned by other ultrastructural CCH studies.

Among the CCH cells, we also consistently observed interdigitations of the cytoplasmic membranes with neighbouring cells and adherens-type cell junctions, as previously reported by our team [34], but not by other works. CCH expresses E-cadherin [35], a transmembrane protein that serves as the primary adhesion molecule in adherens junctions, facilitating the attachment of Langerhans cells to keratinocytes [36,37,38,39]. Despite E-cadherin diminishing during the regression process [12], in our ultrastructural study, we only used tumours in an early stage of regression (Groups I and II), which may explain the consistent presence of these junctions.

The multilamellar structures we observed, some with distinct membranes and others where there is membrane compaction, may correspond to compartments enriched in MHC class II, described in dendritic cells, including Langerhans cells [40,41,42,43]. These compartments also undergo structural changes with maturation, evolving from multilamellar structures (type I), which are compressed, resulting in an intermediate type (type II), and in type III or residual, where the membranes are densely compressed [43]

MHC-II antigens have been identified in Langherhan cells [44] and in canine cutaneous histiocytoma, and their redistribution is thought to be linked to disease regression. MHC was identified on the cell membrane in two main patterns, intracytoplasmic and peripheral [15,19]. These patterns likely reflect different stages of the biosynthesis and organisation of MHC molecules during the immune response mediated by Langerhans cells and dendritic cells. In immature dendritic cells, most MHC-II molecules are located intracellularly within storage vesicles, endosomes, and lysosomes, known collectively as MHC-II compartments. These compartments, also found in Langerhans cells, exhibit various morphologies, including a multivesicular appearance or intermediate forms with internal vesicles and concentric membrane arrays [45]. As dendritic cells mature, similarly to the evolution of CCH cells, MHC-II compartments are reorganised, and MHC-II molecules are effectively transported to the cytoplasmic membrane. The presence of MHC-II molecules on the cell membrane, as observed in human Langerhans cell histiocytosis studies, could signify a T-cell-activating ‘mature’ functional state of CCH, which is important for the initiation and progression of tumour regression [40,43,45,46].

One limitation of this study was using samples fixed in formalin rather than with appropriate fixation for transmission electron microscopy studies.

Canine cutaneous histiocytoma (CCH), the most common tumour in young dogs, is characterised by the proliferation of histiocyte-like cells originating from epidermal dendritic cells and exhibits a spontaneous regression phenomenon. In the era of molecular characterisation of tumours, basic research and structural characterisation of tumours in different phases remain crucial. This approach enhances our understanding of the cellular dynamics and contributes to tumour diagnosis. Despite some limitations in sample fixation, our findings provide valuable insights into CCH and underscore the importance of continued investigation into the histopathological and ultrastructural features of tumours.

## Figures and Tables

**Table 1:** Histopathological parameters evaluated in CCH.

Histopathological Parameters	Group I		Group II		Group III		Group IV		Total	
	n	%	n	%	n	%	n	%	n	%
Ulceration										
Absent	2	13.3	1	6.7	5	11.9	2	9.5	10	10.8
Mild	7	46.7	6	40.0	7	16.7	0	0. 0	20	21.2
Moderate	4	26.7	4	26.7	16	38.1	8	38.1	32	34.4
Extensive	2	13.3	4	26.7	14	33.3	11	52.4	31	33.3
Epidermis										
Normal	1	6.7	0	0.0	4	9.5	2	9.5	7	7.5
Ulcerated	2	13.3	5	33.3	14	33.3	11	52.4	32	34.4
Regular Hyperplasia	2	13.3	0	0.0	2	4.8	1	4.8	5	5.4
Irregular Hyperplasia	2	13.3	2	13.3	6	14.3	4	19.1	14	15.1
Pseudocarcinomatous	8	53.3	8	53.3	16	38.1	3	14.3	35	37.6
Necrosis										
Absent	9	60.0	5	33.3	9	21.4	11	52.4	34	36.6
Sparse	4	26.7	5	33.3	8	19.1	2	9.5	19	20.4
Moderate	2	13.3	4	26.7	18	42.9	6	28.6	30	32.3
Extensive	0	0.0	1	6.7	7	16.7	2	9.5	10	10.8
Necrosis (distribution)										
Peripheral	5	33.3	7	46.7	6	14.3	3	14.3	21	22.6
Difuse	1	6.7	3	20.0	27	64.3	7	33.3	38	40.9
Multinucleated Cells										
Absent	14	93.3	12	80.0	36	85.7	10	47.6	72	77.4
Present	1	6.7	3	20.0	6	14.3	11	52.4	21	22.6
Mitotic count										
<2	0	0.0	1	6.7	5	11.9	14	66.7	20	21.5
2–5	7	46.7	6	40.0	27	64.3	6	28.6	46	49.5
>5	8	53.3	8	53.3	10	23.8	1	4.8	27	29.0
Stroma										
Scarce	15	100	15	100	35	83.3	7	33.3	72	77.4
Moderate	0	0.0	0	0.0	7	16.7	14	66.7	21	22.6
Abundant	0	0.0	0	0.0	0	0.00	0	0.0	0.0	0.0

## Data Availability

Dataset available on request from the authors.
